# Stathmin Protein Level, a Potential Predictive Marker for Taxane Treatment Response in Endometrial Cancer

**DOI:** 10.1371/journal.pone.0090141

**Published:** 2014-02-25

**Authors:** Henrica M. J. Werner, Jone Trovik, Mari K. Halle, Elisabeth Wik, Lars A. Akslen, Even Birkeland, Therese Bredholt, Ingvild L. Tangen, Camilla Krakstad, Helga B. Salvesen

**Affiliations:** 1 Centre for Cancer Biomarkers, Department of Clinical Science, The University of Bergen, Bergen, Norway; 2 Department of Gynecology and Obstetrics, Haukeland University Hospital, Bergen, Norway; 3 Centre for Cancer Biomarkers, Department of Clinical Medicine, The University of Bergen, Bergen, Norway; 4 Department of Pathology, Haukeland University Hospital, Bergen, Norway; IPO, Inst Port Oncology, Portugal

## Abstract

Stathmin is a prognostic marker in many cancers, including endometrial cancer. Preclinical studies, predominantly in breast cancer, have suggested that stathmin may additionally be a predictive marker for response to paclitaxel. We first evaluated the response to paclitaxel in endometrial cancer cell lines before and after stathmin knock-down. Subsequently we investigated the clinical response to paclitaxel containing chemotherapy in metastatic endometrial cancer in relation to stathmin protein level in tumors. Stathmin level was also determined in metastatic lesions, analyzing changes in biomarker status on disease progression. Knock-down of stathmin improved sensitivity to paclitaxel in endometrial carcinoma cell lines with both naturally higher and lower sensitivity to paclitaxel. In clinical samples, high stathmin level was demonstrated to be associated with poor response to paclitaxel containing chemotherapy and to reduced disease specific survival only in patients treated with such combination. Stathmin level increased significantly from primary to metastatic lesions. This study suggests, supported by both preclinical and clinical data, that stathmin could be a predictive biomarker for response to paclitaxel treatment in endometrial cancer. Re-assessment of stathmin level in metastatic lesions prior to treatment start may be relevant. Also, validation in a randomized clinical trial will be important.

## Introduction

Stathmin1 (STMN1 hereafter indicated as ‘stathmin’) is an 18 kD cytosolic phosphoprotein, known to play an important role in the cell cycle. Stathmin is expressed in all tissues. It is a critical regulator of microtubule dynamics through its microtubule destabilizing properties, including both prevention of polymerization and active promotion of microtubule depolymerization [1]–[4]. Phosphorylation of stathmin on four serine residues in the beginning of the mitotic phase attenuates its destabilizing activities, allowing cells to form a mitotic spindle; dephosphorylation then takes place prior to exit of mitosis [1], [4]. Stathmin is also involved in intracellular transport, cell motility, polarity, maintenance of cell shape and regulation of apoptosis [1].

A biomarker is defined as a 'characteristic that is objectively measured and evaluated as an indicator of normal biologic processes, pathogenic processes or pharmacologic responses to a specified therapeutic intervention [5]. Biomarkers can be divided in various types, such as prognostic; linked to the prognosis of a patient independent of treatment, and predictive biomarkers; that identify patient subpopulations most likely to (not) respond to a treatment [5]. Thus, reliable predictive biomarkers are of paramount importance for improved and individualized treatment.

Stathmin is upregulated in many solid cancers, including endometrial cancer [1], [6]–[14], and its upregulation has been associated with clinicopathological variables of aggressive disease such as increased risk of lymph node metastasis [9], [15] and poor survival [6], [9], [10], [12], [13], [16], confirming stathmins role as a prognostic biomarker.

Presently, few predictive markers are known in human cancers and even less are clinically applied. In endometrial cancer no clinically validated predictive markers are yet available [17]. Both targeted therapies and conventional chemotherapeutic agents are effective only in a subset of patients [18], [19], there is therefore an urgent need to identify clinically useful predictive markers. Examples incorporated in the clinic include *KRAS* mutational status indicating response to cetuximab and panitumumab in colorectal cancer [18], [20], [21], ALK re-arrangement in non-small cell lung cancer predicting response to crizotinib [18], [20], [22] and HER2/Neu amplification or overexpression in breast cancer for eligibility for trastuzumab treatment [18], [20], [23].

Taxanes are a group of chemotherapeutic agents frequently used in the treatment of endometrial carcinoma. Preclinical studies in breast and prostate cancer and retinoblastoma [24]–[28] give preclinical indications that stathmin may be a predictive marker for response to taxanes in these cancer types. High levels of stathmin decreased the sensitivity of breast cancer cell lines to paclitaxel and vincristine [24] and knock-down of stathmin by siRNA increased the sensitivity to paclitaxel in both breast [25] and prostate cells [27]. This impact of stathmin protein level on treatment response was limited to anti-microtubule agents. Unfortunately, none of these studies have taken this knowledge to a next level, integrating the results with clinical data. In endometrial cancer to our knowledge no studies, preclinical nor clinical, have explored an association between stathmin level and response to paclitaxel containing chemotherapy. In this report, we demonstrate in endometrial carcinoma cell lines, that reduction of stathmin levels by stathmin knock-down results in improved response to paclitaxel. We also show for the first time to the best of our knowledge, that stathmin protein level is associated with response to paclitaxel containing therapy in clinical samples from patients with metastatic endometrial carcinoma.

## Materials and Methods

### Cell lines

Two endometrial cancer cell lines were selected due to the difference in their sensitivity profile to paclitaxel; Ishikawa (Sigma, sensitive) and Hec1B (American Type Culture Collection, reduced sensitive). The Cancer Cell Line Encyclopedia (CCLE) data confirms the difference in sensitivity [29]. The lines were obtained in 2009 and authenticity verification by short tandem repeat (STR) profiling was performed in 2012 [30], [31]. The cell lines were maintained under the conditions recommended by the suppliers.

### Cell transfection

Cells were cultured to 50–70% confluence prior to transfection by lentiviral transduction (Open biosystems, GIPZ lentiviral shRNAmir). A GIPZ lentiviral shRNA target gene set of 3 (V3LHS_411977; V2LHS_62940; V3LHS_383505) at MOI 2.5 was used. A non-silencing GIPZ lentiviral shRNAmir control (BV17110_2.80×10 8) was used as control. Cells were selected with puromycin (1 μg/ml) after transfection.

### Drugs

Paclitaxel and carboplatin were purchased from Sigma.

### Cell line experiments

The cell lines were treated with paclitaxel in increasing concentrations (range 1–500 nM) for 24 h. As clinically taxanes are often combined with platinum derivates in endometrial cancer, we also treated cells with a combination of paclitaxel (in increasing concentrations (range 1–500 nM) and carboplatin (fixed concentration, 200 mM) for 24 h to observe any synergistic treatment effects. Cells were subsequently either fixed in 2% formaldehyde for microscopic evaluation of apoptosis; used in a proliferation assay (MTS) or processed for immunoblotting. Experiments were at least performed in triplicate.

For assessment of apoptosis, at least 150 cells were counted in three different areas in 96-well plates. For proliferation assays, experiments were performed in triplicates in 96-well plates. Assays were performed with CellTiter 96® AQ_ueous_ One Solution Cell Proliferation Assay (Promega) following instructions from the manufacturer. The absorbance was recorded at 490 nm using an ELISA plate reader (TECAN Magellan Sunrise).

Immunoblots were performed according to a standard protocol. In short, cells were grown and treated in 6-well plates and harvested in lysisbuffer after 24 h paclitaxel treatment. Proteins (25 ug) were separated by SDS/PAGE and transferred to a nitrocellulose membrane (Biorad, Norway). Stathmin and/or (cleaved) PARP were detected using cleaved PARP (Asp214) (D64E10) (#5625Cell Signaling), diluted 1∶1000 and stathmin (#3352, Cell Signaling), diluted 1∶1000; β-actin served as a loading control (anti β-actin antibody (AC-15) (ab6276) AbCam), diluted 1∶10000. Alkaline phosphatase conjugated secondary antibodies were used (Anti-rabbit IgG (Sigma Aldrich A3687): Anti-mouse IgG (Sigma Aldrich A3562)) and chemoluminiscence substrate (lumiphos 34150 WB, Thermo scientific) for detection.

### Patient series

Patients diagnosed with and treated for endometrial cancer at Haukeland University Hospital, Bergen, Norway, are after signing informed consent, prospectively and consecutively included in a database (population based setting) from 2001 onwards, preventing selection bias and ensuring optimal data collection for all patients, as previously reported [14]. Patients have however been treated following routine guidelines and the clinical samples investigated therefore consist of prospectively collected archival tissue. Clinicopathological data collected include amongst others FIGO 2009 stage, histological subtype, grade, primary and adjuvant treatment, and follow up including treatment for metastatic disease. For the purpose of this study, patients who received paclitaxel containing chemotherapy (as a routine in our hospital a combination of paclitaxel and carboplatin) after surgical treatment for either residual disease or metastasis before April 2011, were studied for treatment response according to RECIST criteria [32], with last follow-up entry July 2013. Of in total 607 patients in the database, of which 121 had systemic i.e. recurrent or residual disease, 57 had response data according to RECIST criteria available; 33 of which were treated with paclitaxel containing chemotherapy. We defined good response as complete or partial response (RECIST criteria), and poor response as static disease or disease progression (RECIST criteria). In addition we looked at disease specific survival in relation to stathmin level for all patients with endometrial cancer and specifically for patients treated for metastatic disease. The mean follow-up in our cohort was 34 months (range 0–105 months).

### Tissue microarray (TMA) construction

TMA's were generated as previously described and validated in several studies [33]. The area of highest tumor aggressiveness was identified on all hematoxylin/eosin slides to ensure tumor representativity and three (primary tumor) or one (metastasis) tissue cylinders (0.6 mm diameter each) were mounted in a recipient block using a custom made precision instrument (Beecher instruments, silver spring, MD, USA). Formalin fixed paraffin embedded (FFPE) primary tumor tissue was available in TMAs from 603 patients for evaluation of stathmin level. From 77 patients with metastases, additional metastatic tissue was available in TMAs for investigation of stathmin level compared to the corresponding primary tumor. Too few cases had additional evaluable metastatic lesions, obtained prior to the paclitaxel containing chemotherapy, for stathmin level evaluation, with response data available according to the RECIST criteria and a similar prior treatment profile (n = 3) to allow meaningful statistical analyses of response in relation to biomarker status in metastatic lesions.

### Immunohistochemistry

5 μm thick TMA sections were dewaxed with xylene/ethanol. Antigen retrieval was done by microwave in TRS pH6 (S1699 Dako Target Retrieval Solution) for 20 minutes. Slides were blocked for peroxidase (Dako S2023) for 8 minutes and incubated for 60 minutes with stathmin (Cell signaling #3352), diluted 1∶50. EnVision+ system, HRP secondary antibody (Dako K4011) was used, followed by DAB+chromogen (DAKO K4011) as detection system. Slides were counterstained with hematoxylin (Dako, S2020).

### Staining evaluation

Blinded for patient characteristics and outcome, slides were scored by two authors (HMJW and JT) using standard light microscopy as previously described [34], [35]. The kappa value, as a measure of reproducibility, was 0.73 in a separate set of 68 slides scored individually by HMJW and JT. High protein level was defined as the upper quartile, score 9, in line with previous publications [15]. In case of multiple metastases with variation in stathmin level, the lesion with highest level defined the final score for metastatic lesions.

### Statistics

Statistical analyses were performed using PASW18 Statistics (Predictive Analysis SoftWare, SPSS inc. Chicago, USA). Categorical variables were evaluated using the Pearson χ2-test or Fisher exact where applicable. Two-sided P-values of <0.05 were considered significant. Univariate analyses of time from primary treatment to death due to endometrial carcinoma (disease specific survival) were carried out using the Kaplan-Meier method. The Cox proportional hazards method was used for a multivariate survival analysis (proportionality assumption checked by log minus log plot).

### Ethics statement

All patients have signed informed consent prior to inclusion in the study. The study has been approved by the Norwegian Data Inspectorate (961478-2), the Norwegian Social Science Data Services (15501) and the local Institutional Review Board (Regional Committees for Medical and Health Research Ethics; REKIII nr 052.01).

## Results

### Response to paclitaxel in endometrial cancer cell lines

**Figure 1:**
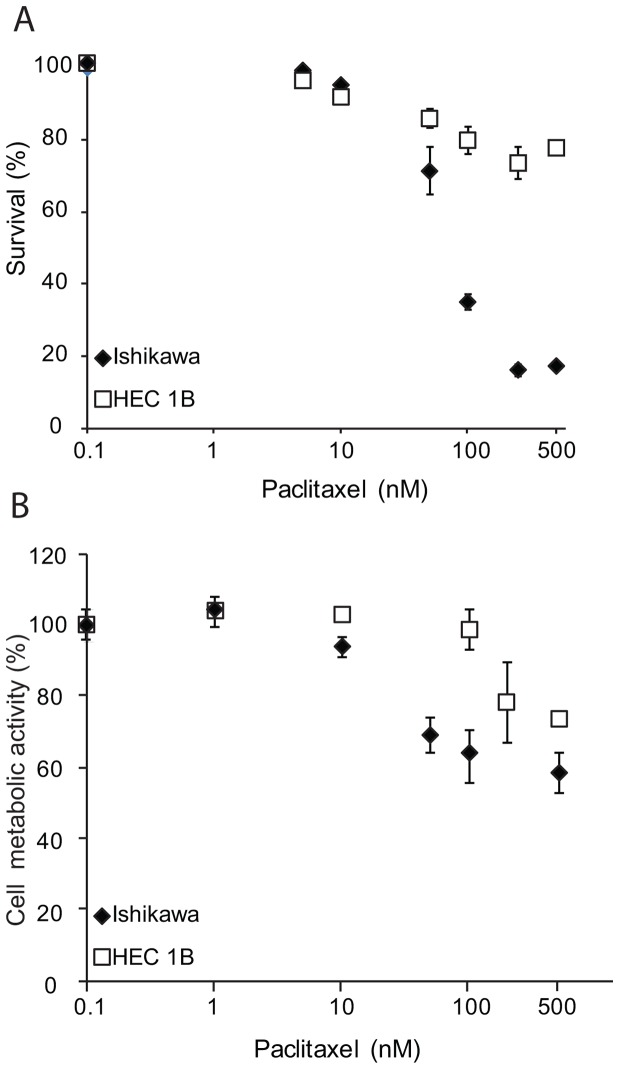
Sensitivity of wild-type cell lines to paclitaxel treatment. A: Microscopic assessment of apoptosis in Ishikawa and Hec1B wild-type cells after treatment with paclitaxel in the following dosages: 0 nM, 5 nM, 10 nM, 50 nM, 100 nM, 250 nM and 500 nM. Results are representative of 5 independent experiments. Standard errors of the mean are indicated. B: Cell metabolic activity assessed with a proliferation assay (MTS, Promega) in Ishikawa and Hec1B wild-type cells after treatment with paclitaxel in the following dosages: 0 nM, 1 nM, 10 nM, 50 nM, 100 nM, 250 nM and 500 nM. Results are representative of 3 (Ishikawa) and 2 (Hec1B) independent experiments.

Response to paclitaxel varies between endometrial cancer cell lines [29], [36], [37]. We show Ishikawa cells are sensitive to paclitaxel treatment with a high percentage of apoptotic cells after 24 h treatment (microscopic counting and proliferation assay) as opposed to Hec1B cells (Fig. 1a and 1b). Combination treatment of carboplatin and paclitaxel did not result in synergistic treatment effect (not shown).

### Stathmin knock-down by viral transfection

**Figure 2:**
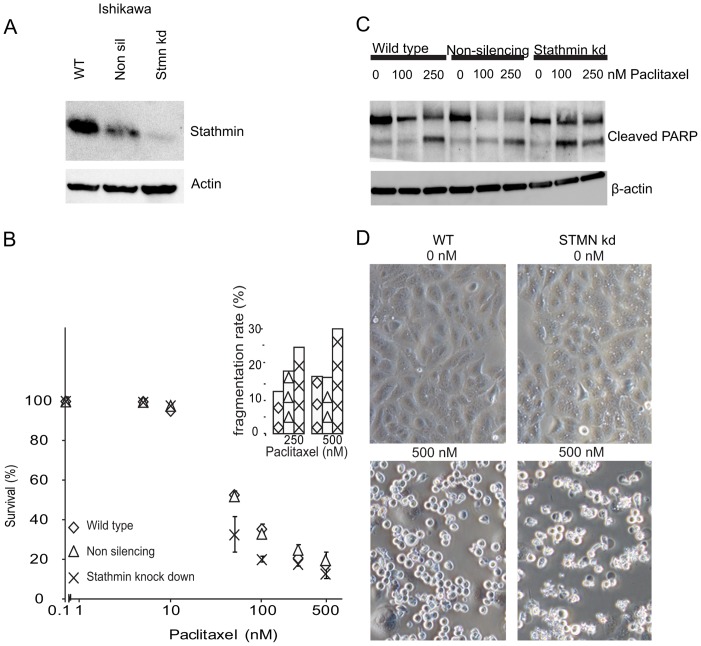
Ishikawa cell line experiments after stathmin knock-down. A: Immunoblot after transfecting cells with a stathmin lentiviral shRNAmir (‘stmn kd’) or a non-silencing control (‘non sil’) as well as the parental cell line (wild-type; ‘WT’). Blots were stained for stathmin, and β-actin for loading control. B: Ishikawa wild-type cell line, non-silencing and stathmin knock-down after treatment with paclitaxel for 24 h in the following dosages: 0 nM, 5 nM, 10 nM, 50 nM, 100 nM, 250 nM and 500 nM. The level of fragmentation of the cells is indicated in an insert, as a proxy of progression in the apoptotic process. Diamonds; wild-type, triangles; non-silencing and crosses; stathmin knock-down cells. C: Immunoblot of Ishikawa wild-type, control (non-silencing) and stathmin knock-down cell lines after treatment with paclitaxel for 24 h in the following dosages: 0 nM, 100 nM and 250 nM. The blot was stained for cleaved PARP and stathmin, with β-actin serving as loading control. D: Left: Ishikawa wild-type (‘WT’) cell line and Right: Ishikawa stathmin knock-down (‘Stmn kd’) cell line. Microscopic images of cells after treatment for 24 h with 0 nM (top row) or 500 nM (bottom row) paclitaxel also demonstrating increased fragmentation rate for the stathmin knock-down Ishikawa cells (right lower panel) compared to wild-type (left lower panel).

**Figure 3:**
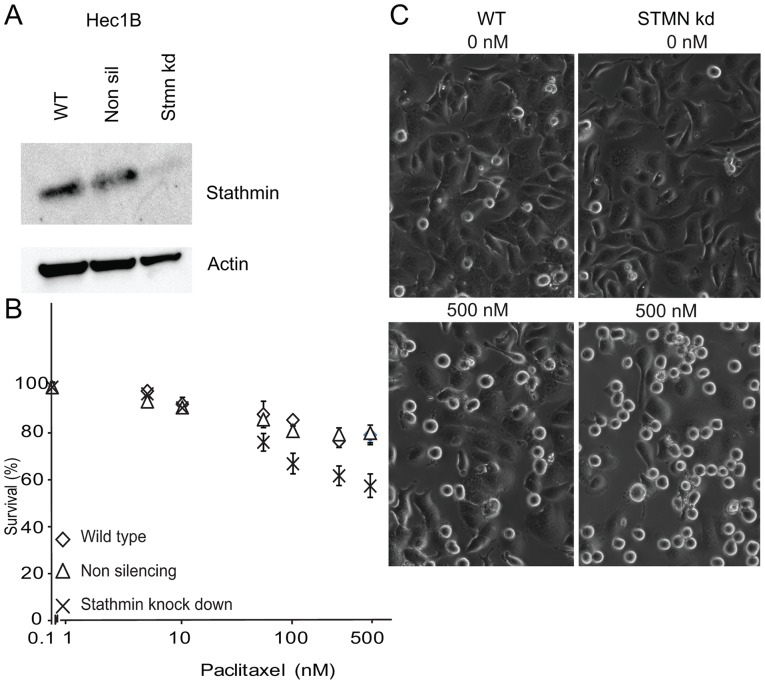
Hec1B cell line experiments after stathmin knock-down. A: Immunoblot after transfecting cells with a stathmin lentiviral shRNAmir (‘stmn kd’) or a non-silencing control (‘non sil’) as well as the parental cell line (wild-type; ‘WT’). Blots were stained for stathmin, and β-actin for loading control. B: Hec1B wild-type cell line, non-silencing and stathmin knock-down lines, after treatment with paclitaxel for 24 h in the following dosages: 0 nM, 5 nM, 10 nM, 50 nM, 100 nM, 250 nM and 500 nM. C: Left: Hec1B wild-type (‘WT’) cell line and Right: Hec1B stathmin knock-down (‘Stmn kd’) cell line. Showing microscopic images of cells after treatment for 24 h with 0 nM (top row) or 500 nM (bottom row) paclitaxel demonstrating increased cell death for the stathmin knock-down Hec1B cells (right lower panel) compared to Hec1B wild-type (left lower panel).

Fluorescence microscopy showed a transfection rate of 70–80% at the start of experiments, with markedly reduced stathmin levels in the stathmin knock-down cell lines compared to the control knock-down and wild-type cell lines (Fig. 2a and 3a).

In both stathmin knock-down cell lines (Ishikawa and Hec1B), improved response to paclitaxel treatment was observed (Fig. 2b and 3b). Hec1B cells show a statistically significant increased apoptotic rate after stathmin knock-down. Possibly due to the intrinsic higher sensitivity to paclitaxel in Ishikawa cells, knock-down did not result in a similar large increase in cell death. However, we noted a clearly increased fragmentation rate in the treated stathmin knock-down Ishikawa cells opposed to the control cells, which may be regarded as a sign of further activation of the apoptotic pathway (insert Fig. 2b). Using immunoblot, we tried to further validate this enhanced apoptotic pathway activation demonstrating PARP cleavage at a lower paclitaxel concentration for Ishikawa after stathmin knock-down compared to controls (Fig. 2c). Microscopic pictures of Ishikawa and Hec1B wild-type and stathmin knock-down cells after 24 h paclitaxel treatment with 0 nM (control) and 500 nM are shown in Figures 2d and 3c. We tested the effect of stathmin knock-down on the sensitivity to carboplatin monotherapy and paclitaxel-carboplatin combinational treatment without observing increased sensitivity or synergistic effects (not shown).

### High stathmin level predicts poor response to paclitaxel in clinical samples

**Figure 4:**
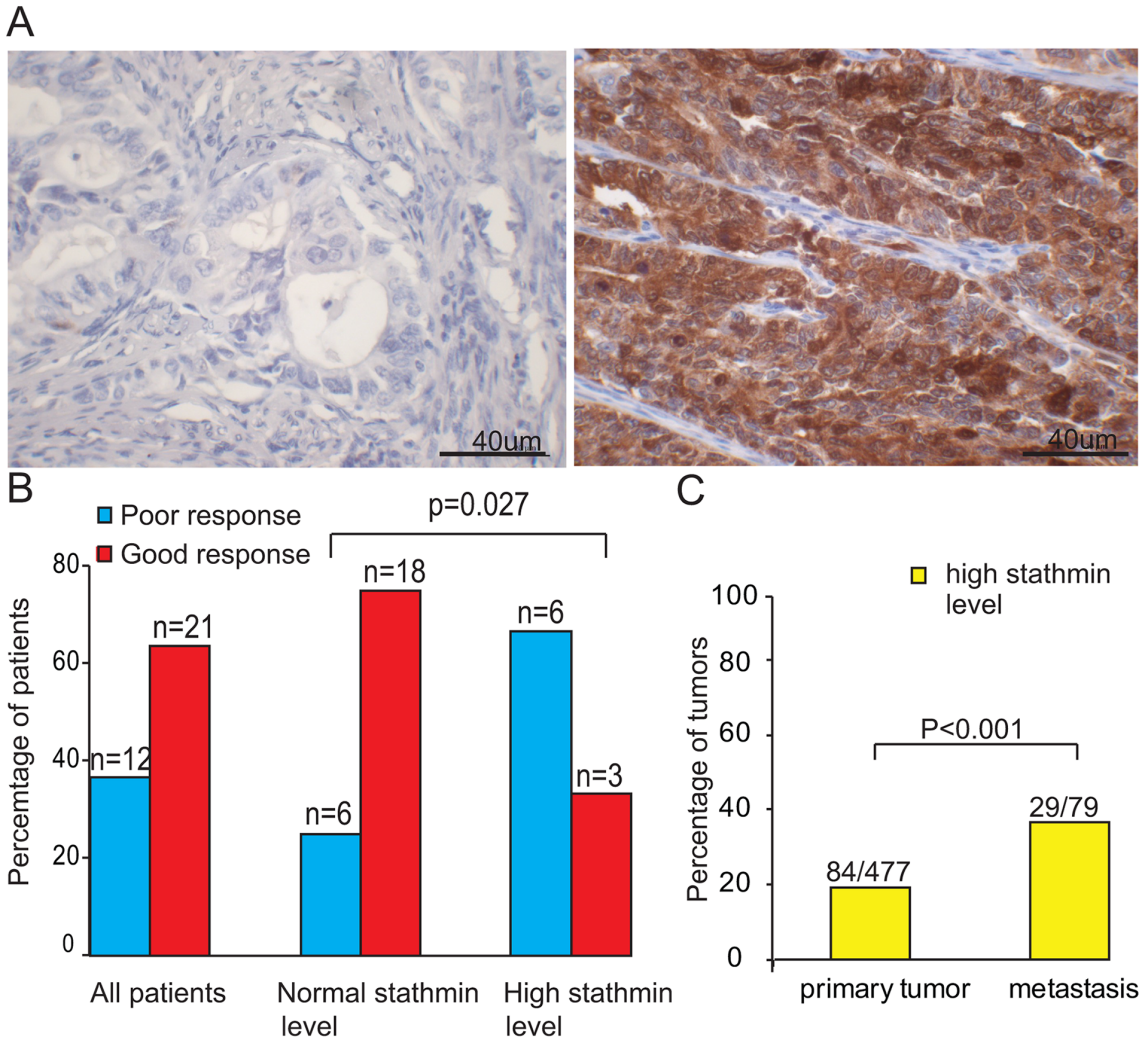
Stathmin protein expression in relation to clinical parameters. A: Pictures representative of weak immunohistochemical stathmin staining (top) and strong (high or pathologic) stathmin staining (bottom) in endometrial carcinoma. Bars (right lower corner) measure 40 μm. B: Clinical response to paclitaxel for all endometrial carcinoma patients with evaluable disease according to RECIST criteria and separated for normal versus high stathmin level. Poor response (RECIST: static disease or disease progression) indicated in blue, good response (RECIST: complete or partial response) indicated in red. C: Comparison of high (pathologic) stathmin protein level in primary and metastatic lesions.

We then investigated patient tumor samples to see if a similar association between stathmin level and treatment response could be observed. Stathmin staining was predominantly cytoplasmic, as reported in literature [15], [38]. Representative pictures from immunohistochemistry with weak (normal) and strong (high) stathmin staining are shown in Figure 4a. Excluding metastatic patients receiving anti-hormonal treatment only, patients with metastatic disease receiving paclitaxel containing chemotherapy had similar clinicopathological characteristics as patients treated differently. Including the patients treated with anti-hormonal drugs only, predominantly frail elderly patients, clinicopathological characteristics still remained similar, except that this subgroup was significantly older (Table 1). Patients with normal stathmin level clearly responded much better (RECIST criteria) to treatment than patients with high stathmin level (Fig. 4b). Stathmin level did not predict response to other chemotherapy regimens or treatment modalities.

**Table 1:** Characteristics of patients receiving paclitaxel or other treatment for metastatic endometrial cancer (n = 78).

Variable		Paclitaxel n (%)	Other treatment n (%)	P-value	
FIGO					0.712
	I/II	5 (22.7)	15 (26.8)		
	III/IV	17 (72.3)	41 (73.2)		
Histology					0.765
	Endometrioid	13 (59.1)	31 (55.4)		
	Non-endometrioid	9 (40.9)	25 (44.6)		
Histological differentiation^1^					0.365
	I/II	6 (27.3)	21 (38.2)		
	III	16 (72.7)	34 (61.8)		
Age (median 66)					0.031
	Below/equal to	15 (68.2)	23 (41.1)		
	Above	7 (31.8)	33 (58.9)		
Menopausal status					0.255
	Pre/perimenopausal	3 (13.6)	3 (5.4)		
	Postmenopausal	19 (86.4)	53 (94.6)		
Stathmin expression^2^					0.891
	Normal	15 (71.4)	37 (69.8)		
	High expression (9)	6 (28.6)	16 (30.2)		

^1^ information missing for 1 patient.

^2^ information missing for 4 patients.

**Figure 5:**
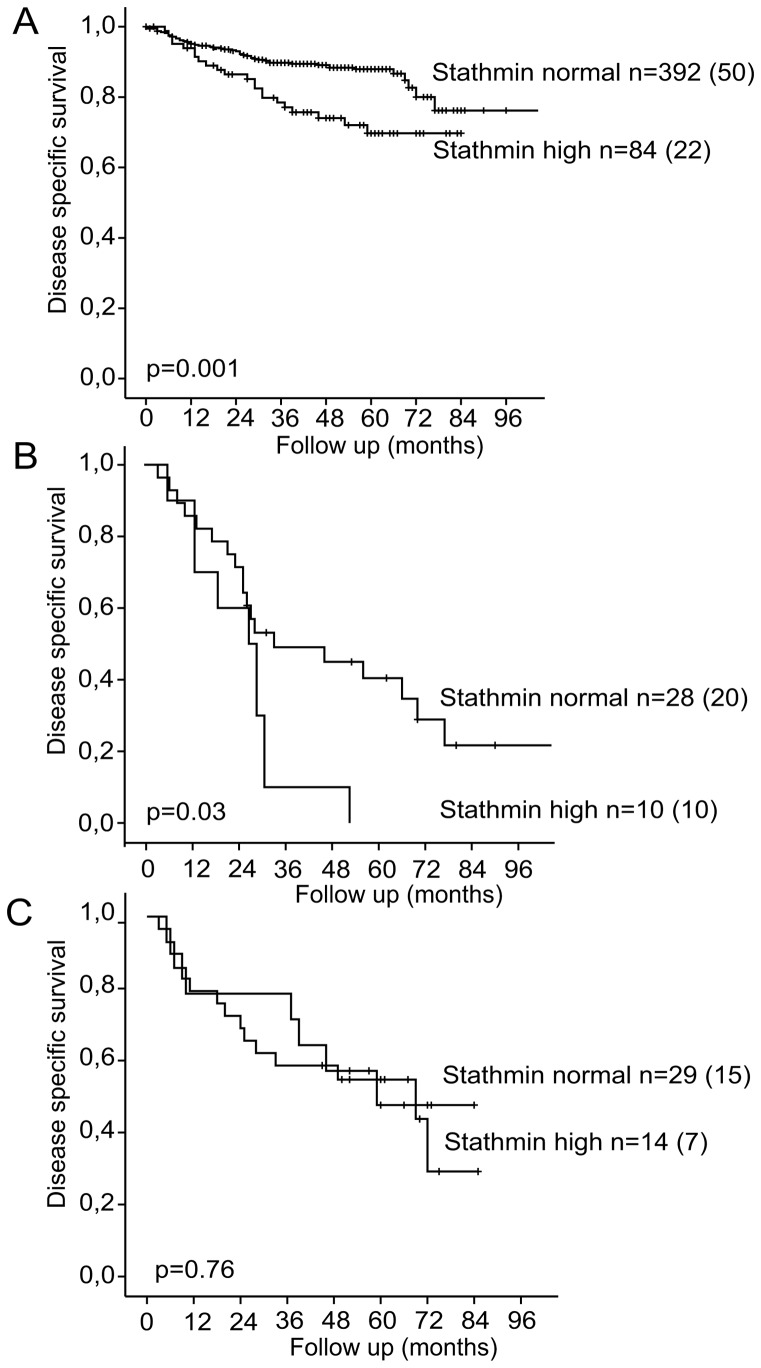
Disease specific survival after primary treatment for endometrial carcinoma patients (Kaplan-Meier curves) related to stathmin protein expression by IHC in primary tumor. A: All patients with complete data (n = 476). Number of disease specific events between brackets. B: All patients with metastatic disease who received paclitaxel treatment (n = 38). Number of disease specific events between brackets. C: All patients with metastatic disease who received different treatments (n = 43). Number of disease specific events between brackets.

Approaching from a different angle, in general, patients with high stathmin level showed a reduced disease specific survival, in line with stathmins role as a prognostic biomarker (Fig. 5a). However, within the subgroup of patients with metastatic disease treated with paclitaxel containing chemotherapy, disease specific survival was significantly poorer in those patients with high compared to normal stathmin (p = 0.03, Fig. 5b). In patients who received other treatments for metastatic disease, prognosis was unrelated to stathmin level (p = 0.76, Fig. 5c. To rule out confounding by known important clinicopathological prognostic variables, we performed a multivariate survival analysis for both subgroups to look into the effect of stathmin level on survival after treatment for metastatic disease, corrected for FIGO stage and histological subtype. Stathmin protein level remained an independent predictor of disease specific survival in the subgroup of patients that received paclitaxel containing chemotherapy (n = 38, HR 2.3, CI 1.1–5.2), adjusted for FIGO stage and histological subtype, but not in the subgroup receiving other therapies (n = 43, HR 1.1, CI 0.4–2.7).

### Discordant biomarker status in primary and metastatic lesions

The percentage of patients with high stathmin level was significantly higher in metastases compared to primary lesions with pathologic (high) levels noted in 18% of the latter (n = 84 of 477 primary lesions with stathmin staining available) compared to 37% in metastatic samples (n = 29 of 79) (Fig. 4c).

In the paired primary-metastasis samples, 35% of metastatic lesions showed high stathmin level. A discordance of 26% between metastatic lesions and their primaries was observed. In 16% there was a change to high level in metastases and in 10% to normal level.

## Discussion

Stathmin protein level has been shown to be a prognostic marker of aggressive disease in many cancers, including endometrial cancer, where high stathmin level in primary tumor identifies patients at high risk for recurrent disease and lymph node metastases [6], [9], [10], [12], [13], [15], [16]. The identification and development of predictive biomarkers are of paramount importance to increase treatment efficacy and reduce unnecessary side effects, not only in targeted therapies but also in chemotherapeutic regimes, as for both counts that only a subpopulation will respond well, especially in the metastatic setting, but with currently very limited tools available to predict these patients [39], [40]. None of the important clinicopathological factors, such as FIGO stage or histological subtype, are currently known to help distinguish potential responders from non-responders to paclitaxel containing chemotherapy in the metastatic setting. Studying large population based series with high-quality clinical annotation such as our series, combined with preclinical experiments are a useful and time-efficient tool to explore potential predictive biomarkers, which can subsequently be tested in clinical trials.

In line with previous *in vitro* results in breast cancer, we show in endometrial cancer cell lines that, independent of the original stathmin level, sensitivity to paclitaxel increased and thereby apoptosis expedited after successful stathmin knock-down. This was shown by direct microscopic counting and in Ishikawa cells also substantiated by immunoblotting focusing on PARP cleavage. PARP cleavage is an established indicator of apoptosis, distinguishing it from other mechanisms of cell death, such as necrosis. The increased apoptotic body formation noted by microscopy in the stathmin knock-down cell lines fits with increased apoptosis [41], [42].

In our prospectively collected, retrospectively analyzed patient series, we also demonstrated a striking difference in response to paclitaxel containing chemotherapy comparing patients with normal to those with high stathmin level, also when correcting for the most important clinicopathological prognostic variables. Even when exploring such a large clinical series with endometrial cancer patients as ours, collected over more than 10 years, with adequate follow-up and RECIST [32] compliant documentation of response, ultimately only a smaller number of patients had been treated with the treatment of interest, underlining the difficulty of collecting series with adequate patient numbers for specific marker studies; but at the same time the importance to exploit these large prospectively collected population based series for predictive biomarkers suggested in preclinical studies, and explore potential clinical validity prior to clinical trial stage. The statistically significant correlation between high stathmin level and poor paclitaxel response according to RECIST criteria in clinical samples and the fact that stathmin level has an independent prognostic value in patients receiving paclitaxel for metastatic disease, not present in patients who do not, in survival analyses, supports the likelihood that the level of stathmin level may act not only as a prognostic marker but also as a predictive marker for response to paclitaxel treatment in endometrial carcinomas.

Unlike previous studies looking at stathmin as a potential predictive marker, predominantly in *in vitro* breast cancer studies, in this study we were able to test and confirm the association in clinical samples from patients treated with the drug of interest; using data from a well-annotated prospectively collected patient series. Both the preclinical and clinical testing support that stathmin level influences sensitivity to paclitaxel. We have explored and excluded that this effect can be generalized to other chemotherapeutic agents such as carboplatin, also frequently used in endometrial cancer.

Reporting recommendations for tumor marker prognostic studies (REMARK) guidelines have been developed with the aim to improve the methodological quality and reporting transparency in such studies [43]. The current study has been performed in accordance to these guidelines to improve the quality and general validity of its results.

Taxanes, originally isolated from the bark of the yew tree, belong to the family of anti-microtubule chemotherapeutic agents, with paclitaxel as their prototype. Simply put, taxanes bind to β -tubulin, causing microtubules to resist depolymerization, inhibiting cell cycle progression and promoting mitotic arrest and cell death [44]. Carboplatin, in contrast, is one of the platinum based agents, interacting with DNA and interfering with DNA repair. As stathmin is a critical regulator of microtubule dynamics, taken into consideration the mode of action of the drugs, the positive effect of stathmin knock-down on paclitaxel response and the absence of it to carboplatin sensitivity, is also biologically plausible.

We show a higher proportion of high stathmin level in metastatic (37%) compared with primary lesions (18%). Discrepancy in stathmin status was noted in a quarter of paired samples, paralleling findings in e.g. breast cancer where discrepancies between primary and metastatic lesions are shown in 14–55% and 0–40% for hormone receptors and HER2 respectively [45]–[47]. In endometrial cancer, few studies discuss differences in marker status between primary and metastatic lesions [38], [48], [49]. Intratumoral heterogeneity is well described in cancer and a potential confounding factor in many studies, irrespective of using full-tissue slides or TMA. Inter-observer variation is unlikely to be the sole explanation for these described differences. Also, a recent study assessing mutation status, a method considered less subjective than immunohistochemical scoring, in multiple metastatic lesions from one patient with renal cell carcinoma, support that detected biomarker changes from primary to metastatic lesions are real and may be related to and relevant for tumor progression [39]. The changes in biomarker status from primary to metastatic lesions support the need for repeated biopsies in metastatic lesions, to better relate therapy response to potential predictive biomarkers but also to only offer therapies with likely positive effect when predictive biomarkers are available [47], [50], [51]. For breast cancer, The American society of clinical oncology (ASCO) advised in 2007 already that for hormone receptor status, testing should be considered to be repeated in metastatic disease if the results were to influence patient management [52].

## Conclusion

These results, including preclinical data and for the first time data from clinical samples, support that stathmin may be a predictive biomarker for the response to paclitaxel treatment in endometrial cancer. However, confirmatory studies, ideally from randomized clinical trials are needed. The biomarker discordance on tumor progression is in line with other studies on tumor biomarker heterogeneity and supports the need for repeated biopsy in metastatic disease.
